# Comparison of robotic-assisted and laparoscopic-assisted surgery in the treatment of children with Hirschsprung's disease: a systematic review and meta-analysis

**DOI:** 10.3389/fped.2025.1638198

**Published:** 2025-08-06

**Authors:** Zikang Li, Wanfu Li, Haojun Wang, Mengxue Xu

**Affiliations:** Department of Pediatric General Surgery, The First Affiliated Hospital of Xinjiang Medical University, Urumqi, Xinjiang, China

**Keywords:** Hirschsprung's disease, robotic-assisted surgery, laparoscopic-assisted surgery, postoperative complications, children

## Abstract

**Background:**

This study aims to systematically evaluate the differences between robotic-assisted surgery (RAS) and laparoscopic-assisted surgery (LAS) in intraoperative parameters, postoperative complications, and prognostic outcomes for children with Hirschsprung's disease (HSCR). By conducting a meta-analysis, evidence-based insights for clinical practice were sought.

**Methods:**

Following PRISMA guidelines, PubMed, Embase, and Web of Science were searched up to May 10, 2025, to identify comparative studies of RAS and LAS for HSCR. Two reviewers independently screened literature and assessed quality using the Newcastle-Ottawa Scale (*N*OS). RevMan5.4 and STATA18 were used to calculate odds ratios (ORs) and 95% confidence intervals (CIs), with heterogeneity and publication bias evaluated.

**Results:**

Six studies involving 789 children (352 RAS, 437 LAS) were included. Meta-analysis showed significantly less intraoperative blood loss in the RAS group (OR = −6.45, 95%CI: −11.77 to −1.14, *P* = 0.02) but longer operative duration (OR = 19.74, 95%CI: 1.75–37.72, *P* = 0.03). No significant group differences were found in postoperative enterocolitis (OR = 0.66, 95%CI: 0.43–1.01, *P* = 0.06), anastomotic complications (OR = 0.71, 95%CI: 0.35–1.45, *P* = 0.35), soiling (OR = 0.79, 95%CI: 0.39–1.60, *P* = 0.51), adhesive intestinal obstruction (OR = 1.56, 95%CI: 0.22–11.32, *P* = 0.66), wound infection (OR = 0.77, 95%CI: 0.19–3.01, *P* = 0.70), incisional hernia (OR = 1.13, 95%CI: 0.20–6.40, *P* = 0.89), perianal infection (OR = 0.70, 95%CI: 0.40–1.23, *P* = 0.22), urinary retention (OR = 0.23, 95%CI: 0.01–3.59, *P* = 0.29), or gastrointestinal function recovery time (OR = −1.27, 95%CI: −3.70–1.15, *P* = 0.30). Hospital stay was significantly shorter in the RAS group (OR = −0.39, 95%CI: −0.69–−0.10, *P* = 0.009). Egger's test and funnel plot analysis indicated no significant publication bias (*P* = 0.987).

**Conclusions:**

RAS confers advantages in reducing intraoperative blood loss and shortening hospital stay, although it is associated with a longer operative duration. However, no significant difference in the incidence of postoperative complications was noted between RAS and LAS, a finding potentially attributable to the limited sample size. Furthermore, the currently elevated treatment cost of RAS may impede its widespread adoption due to economic limitations. Consequently, large-sample, multicenter randomized controlled trials with extended follow-up periods are warranted to validate long-term outcomes and conduct in-depth investigations into cost-effectiveness.

**Systematic Review Registration:**

PROSPERO CRD420251051595.

## Introduction

Hirschsprung's disease (HSCR) is a rare congenital intestinal malformation characterized by the absence of ganglion cells in the distal rectal wall, often extending proximally. This leads to functional intestinal obstruction due to dysmotility, manifesting as intractable constipation, abdominal distension, and cyclic vomitingHSCR affects approximately 1 in 5,000 live births, with a male-to-female ratio of 4:1 (1–3). Classic HSCR subtypes are classified as short-segment or long-segment types, with the latter including rare variants like total colonic aganglionosis (4). Surgical management of HSCR primarily involves pull-through procedures, such as open surgery, transanal approaches, laparoscopic-assisted surgery (LAS), and robotic-assisted surgery (RAS). The sagittal posterior approach is reserved for complex cases with abdominal adhesions, such as recurrent disease requiring redo resection or stricture repair. RAS, enabled by platforms like the da Vinci system, has gained traction in adult urology (5), gynecology (6), cardiothoracic surgery (7), head and neck surgery (8), and gastrointestinal/hepatobiliary surgery (9) due to its 3D high-definition vision and robotic arm flexibility. Building on adult experience, RAS in pediatric surgery has shifted from experimental trials to clinical use, focusing on complex abdominal and pelvic procedures (10). RAS is now used in pediatric gastroenterology, urology, and cardiothoracic surgery (11). In 2011, Hebra et al. (12) first described robotic Swenson surgery for pediatric HSCR, using an umbilical incision to deploy robotic arms for intestinal pull-through, which established a precedent for robotic technology in congenital gastrointestinal malformations. By 2019, miniaturized robotic instruments and growing pediatric experience enabled multi-center clinical studies and long-term follow-up data on RAS for HSCR. However, whether RAS offers advantages over LAS in intraoperative parameters and outcomes remains unclear, as high-quality meta-analyses are lacking. Guided by PRISMA guidelines (13), this study systematically screened and assessed literature to include all eligible comparative studies of RAS and LAS for HSCR. Through meta-analysis, we aimed to systematically evaluate RAS advantages in intraoperative and prognostic outcomes, providing evidence for clinical decisions in pediatric HSCR management.

## Methods

PROSPERO registration number: CRD420251051595. The PICO framework guided the search: ① Participants: pediatric HSCR patients; ② Intervention: robotic-assisted surgery (RAS); ③ Comparison: laparoscopic-assisted surgery (LAS); ④ Outcomes: intraoperative parameters, prognostic outcomes, and hospital stay. Literature searches in English were performed across PubMed, Embase, and Web of Science up to May 10, 2025. Search terms included “Robotic,” “Laparoscopic,” and “Hirschsprung disease.” The PubMed search strategy was as follows: (“Hirschsprung Disease"[MeSH Terms] OR “Hirschsprung's Disease"[Title/Abstract] OR “Congenital Megacolon"[Title/Abstract] OR “Aganglionic Megacolon"[Title/Abstract] OR “Hirschsprung"[Title/Abstract] OR “Hischsprung"[Title/Abstract]) AND (“Robotic Surgical Procedures"[MeSH Terms] OR “Robotic-Assisted Surgery"[Title/Abstract] OR “Robot-Assisted"[Title/Abstract] OR “Robotic*"[Title/Abstract] OR “Da Vinci"[Title/Abstract] OR “Da Vinci Xi"[Title/Abstract] OR “RALS"[Title/Abstract]) AND (“Laparoscopy"[MeSH Terms] OR “Laparoscopic Surgery"[Title/Abstract] OR “Minimally Invasive Surgery"[Title/Abstract] OR “MIS"[Title/Abstract] OR “Laparoscop*"[Title/Abstract] OR “Minimally-Invasive"[Title/Abstract]). For Web of Science: TS = (“hirschsprung*” OR “hischsprung” OR “congenital megacolon” OR “aganglionic megacolon”) AND TS = (“robot-assisted” OR robotic* OR “robotic surgical procedure” OR “robotic-assisted surgery” OR “da vinci” OR “da vinci xi” OR RALS) AND TS = (“laparoscopic surgery” OR laparoscop* OR laparoscopy OR “minimally invasive surgery” OR MIS OR “minimally-invasive”). For Embase: (‘hirschsprung disease'/exp OR ‘congenital megacolon':ti,ab OR ‘aganglionic megacolon':ti,ab OR ‘hirschsprung*':ti,ab OR ‘hischsprung':ti,ab) AND (‘robot assisted surgery'/exp OR ‘robotic surgical procedure':ti,ab OR ‘robot-assisted':ti,ab OR robotic*:ti,ab OR ‘da vinci':ti,ab OR ‘da vinci xi':ti,ab OR RALS:ti,ab) AND (‘laparoscopy'/exp OR ‘laparoscopic surgery':ti,ab OR ‘minimally invasive surgery':ti,ab OR MIS:ti,ab OR laparoscop*:ti,ab OR ‘minimally-invasive':ti,ab).

### Inclusion and exclusion criteria

#### Inclusion criteria

Eligibility criteria included: ① Comparative studies of RAS and LAS for HSCR; ② RAS as the intervention and LAS as the control; ③ Studies published up to May 10, 2025; ④ HSCR diagnosis confirmed by preoperative imaging or biopsy.

#### Exclusion criteria

① Reviews, case reports, books, guidelines, editorials, or dissertations; ② Duplicate publications or studies with incomplete data.

### Selection process

Three researchers (HJW, ZKL and WFL) independently reviewed titles and abstracts of the records and discussed inconsistencies until consensus was obtained. Then, in pairs, the researchers independently screened the titles and abstracts of all articles retrieved. In case of disagreement, the discussion reached a consensus on which articles, the full text should be screened. Two researchers (ZKL and MXX) independently screened full-text articles for inclusion. Again, in case of disagreement, consensus was reached on inclusion or exclusion by discussion, and if necessary, the third researcher (WFL) was consulted.

### Data extraction and management

The extracted data comprised: authors, year of publication, study type, country, total sample size, intraoperative indicators (blood loss, operative duration), prognostic outcomes (enterocolitis, soiling, anastomotic complications [stricture, fistula, rectovaginal fistula], adhesive intestinal obstruction, constipation, wound infection, incisional hernia, perianal infection, recurrence, urinary retention, gastrointestinal function recovery time), and length of hospital stay. Discrepancies among researchers were resolved by a third researcher. Soiling is defined as the presence of feces or fecal stains on diapers or underwear postoperatively, with the patient unaware of defecation. Recovery of gastrointestinal function is defined as the time to resume postoperative flatus and defecation. The observation period for soiling and gastrointestinal function recovery extends to the first occurrence of the aforementioned conditions during hospitalization.

### Methodological quality assessment

Two reviewers independently assessed study quality using the Newcastle-Ottawa Scale (NOS).

### Statistical analysis

Statistical analyses were conducted using RevMan5.4 and STATA18. Odds ratios (ORs) and 95% confidence intervals (CIs) were calculated for postoperative complications (14). Statistical significance was set at *P* < 0.05 (15). Heterogeneity was evaluated using the I^2^ statistic: random-effects models were applied when *I*^2^ > 50%, and fixed-effects models for *I*^2^ ≤ 50% (16). Publication bias was assessed using Egger's test and funnel plot analysis (17–19).

## Results

### Literature selection and quality assessment

**Figure 1:**
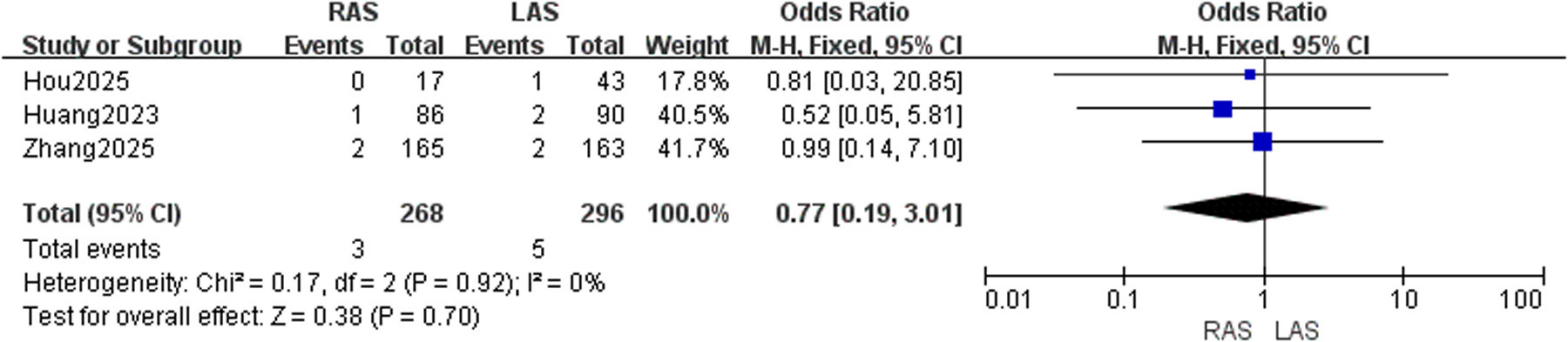
Literature screening flowchart.

A total of 57 publications were retrieved through the search strategy, with 32 duplicates removed. Titles and abstracts of the remaining 25 studies were screened, followed by full-text review of 14 studies. After excluding 8 studies, six studies involving 789 children were ultimately included (Figure 1) (20–25). Basic characteristics and quality assessments are presented in Supplementary Table S1.

### Intraoperative metrics

#### Blood loss

**Figure 2:**
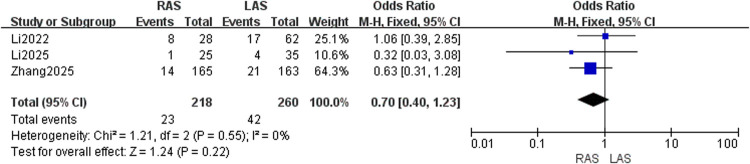
Forest plot of intraoperative blood loss.

Six studies (20–25) including 789 children (352 in RAS group, 437 in LAS group) were included. RevMan5.4 analysis identified high heterogeneity (*I*^2^ = 98%, *P* < 0.00001), requiring a random-effects model. Significantly lower intraoperative blood loss was observed in the RAS group compared to the LAS group (OR = −6.45, 95%CI: −11.77 to −1.14, *P* = 0.02) (Figure 2).

#### Operative duration

**Figure 3:**
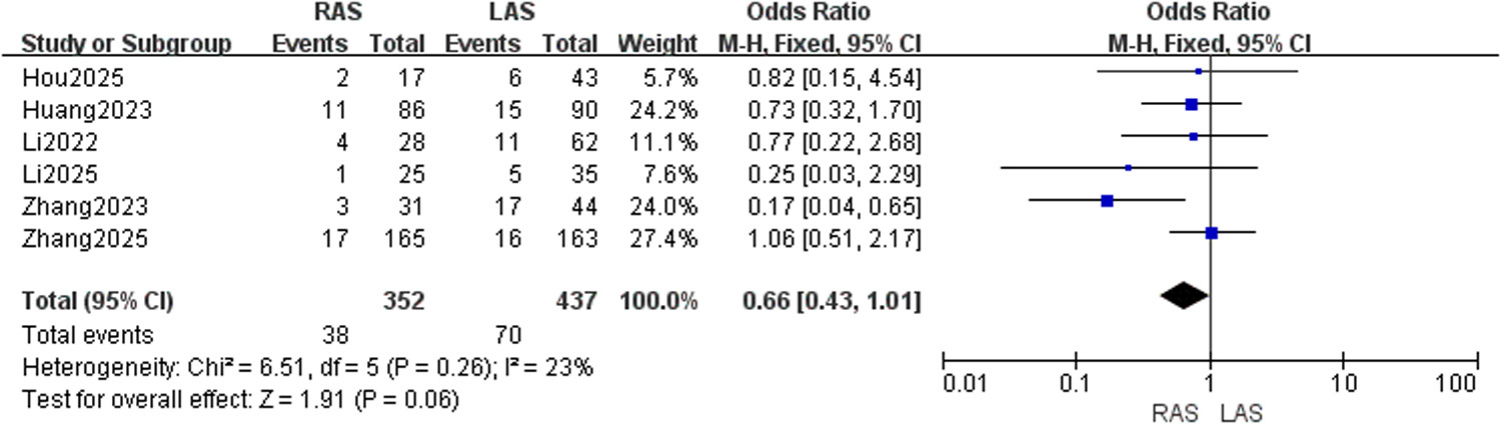
Forest plot of operative duration.

Data from six studies (20–25) were pooled (352 RAS, 437 LAS). Random-effects model analysis demonstrated high heterogeneity (*I*^2^ = 95%, *P* < 0.00001), with significantly longer operative duration in the RAS group (OR = 19.74, 95%CI: 1.75–37.72, *P* = 0.03) (Figure 3).

### Postoperative complications

#### Postoperative enterocolitis incidence

**Figure 4:**
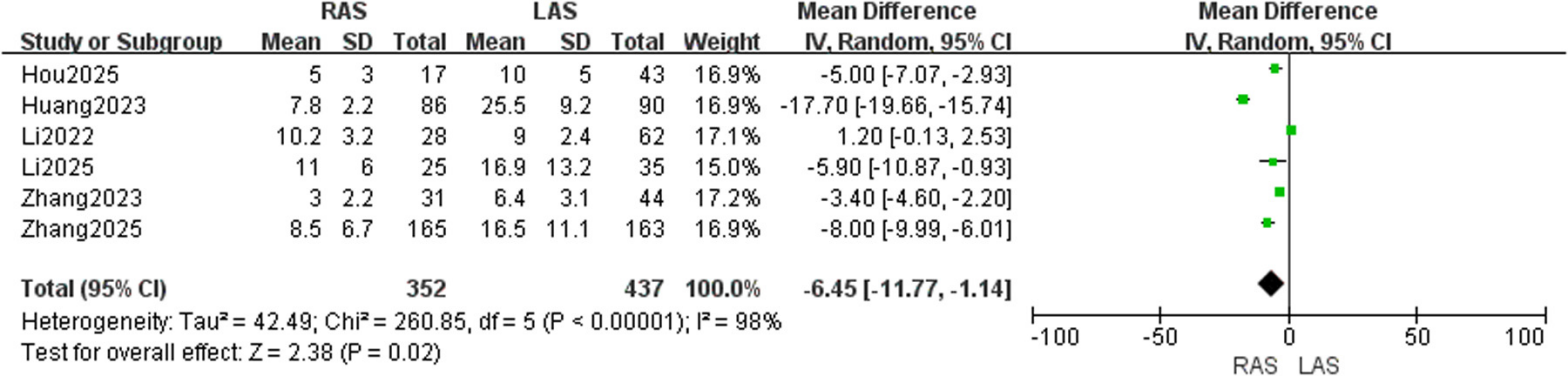
Forest plot of postoperative enterocolitis incidence.

Six studies (20–25) with 352 RAS and 437 LAS patients were analyzed. A fixed-effects model was employed due to low heterogeneity (*I*^2^ = 23%, *P* = 0.26). No significant intergroup difference in enterocolitis incidence was detected (OR = 0.66, 95%CI: 0.43–1.01, *P* = 0.06) (Figure 4).

#### Anastomotic complications

**Figure 5:**
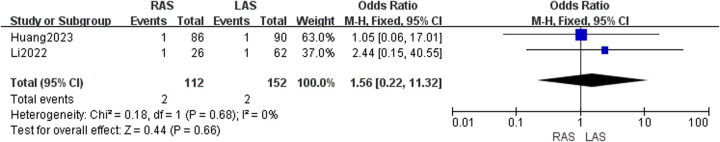
Forest plot of anastomotic complications.

Pooled data from six studies (20–25) demonstrated low heterogeneity (*I*^2^ = 6%, *P* = 0.38). No significant difference in anastomotic complication rates was detected between RAS and LAS (OR = 0.71, 95%CI: 0.35–1.45, *P* = 0.35) (Figure 5).

#### Postoperative soiling rate

**Figure 6:**
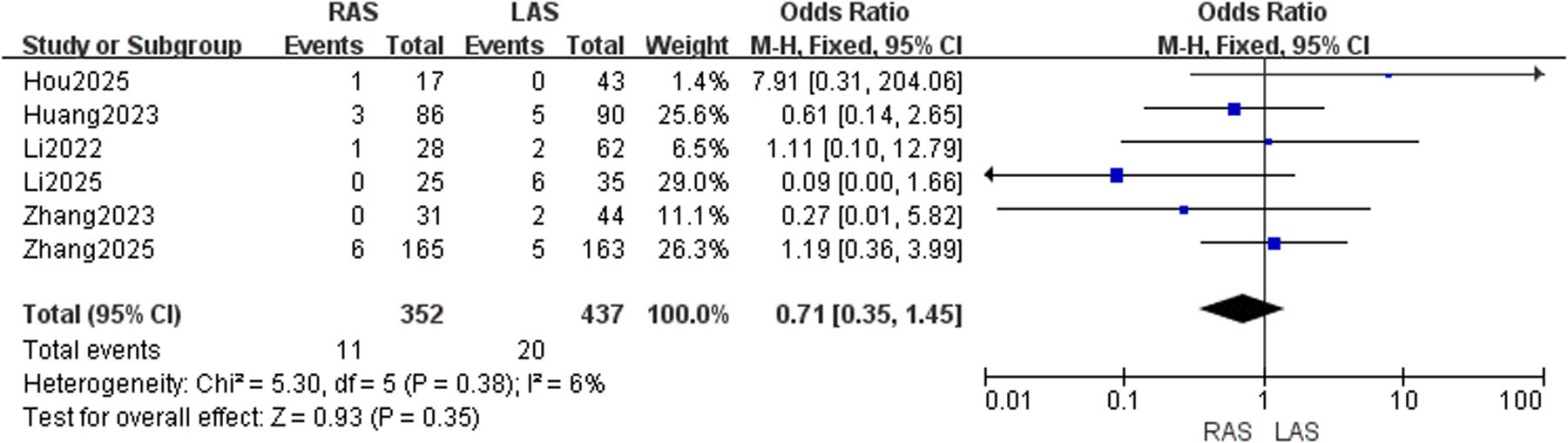
Forest plot of postoperative soiling rate.

Three studies (22, 24, 25) reported soiling in 131 RAS and 195 LAS patients. Fixed-effects model analysis (*I*^2^ = 0%, *P* = 0.75) showed no significant group difference (OR = 0.79, 95%CI: 0.39–1.60, *P* = 0.51) (Figure 6).

#### Adhesive intestinal obstruction

**Figure 7:**
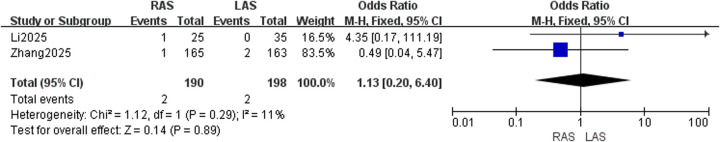
Forest plot of adhesive intestinal obstruction.

Two studies (24, 25) included 112 RAS and 152 LAS patients. Fixed-effects model (*I*^2^ = 0%, *P* = 0.68) showed no significant difference in obstruction rates (OR = 1.56, 95%CI: 0.22–11.32, *P* = 0.66) (Figure 7).

#### Wound infection rate

**Figure 8:**
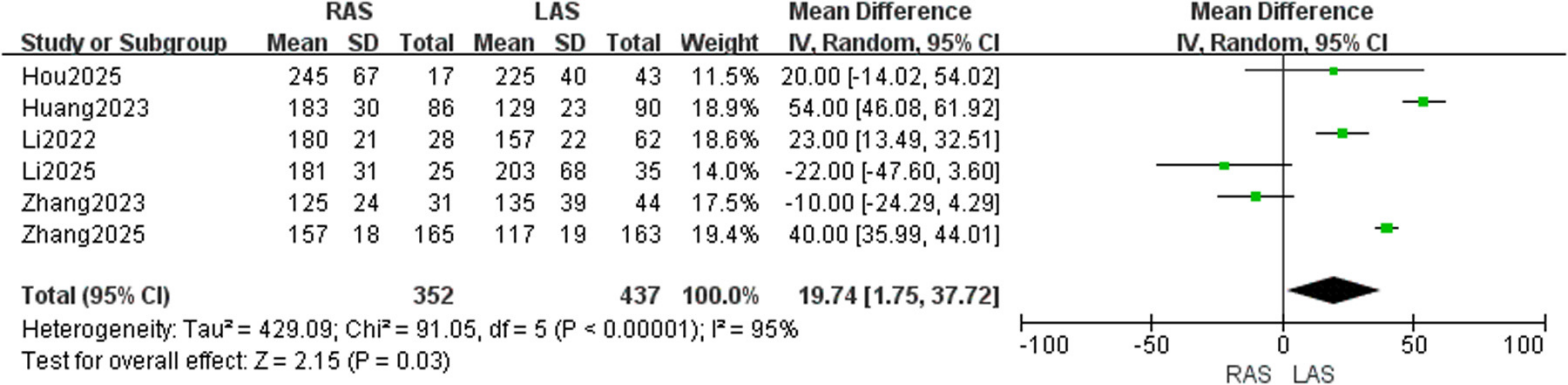
Forest plot of wound infection rate.

Three studies (20, 22, 24) evaluated 268 RAS and 296 LAS patients. Fixed-effects model (*I*^2^ = 0%, *P* = 0.92) showed no significant difference in wound infection rates (OR = 0.77, 95%CI: 0.19–3.01, *P* = 0.70) (Figure 8).

#### Incisional hernia incidence

**Figure 9:**
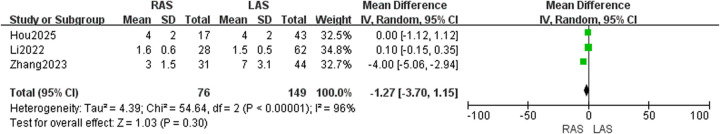
Forest plot of incisional hernia rate.

Two studies (20, 21) included 190 RAS and 198 LAS patients. Fixed-effects model (*I*^2^ = 11%, *P* = 0.29) revealed no significant difference (OR = 1.13, 95%CI: 0.20–6.40, *P* = 0.89) (Figure 9).

#### Perianal infection rate

**Figure 10:**
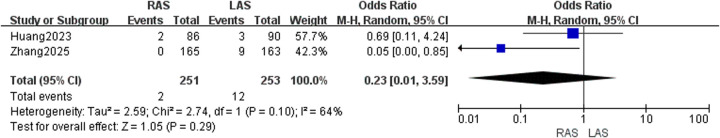
Forest plot of perianal infection rate.

Three studies (20, 21, 25) included 218 RAS and 260 LAS patients. Fixed-effects model (*I*^2^ = 0%, *P* = 0.55) showed no significant difference (OR = 0.70, 95%CI: 0.40–1.23, *P* = 0.22) (Figure 10).

#### Urinary retention rate

**Figure 11:**
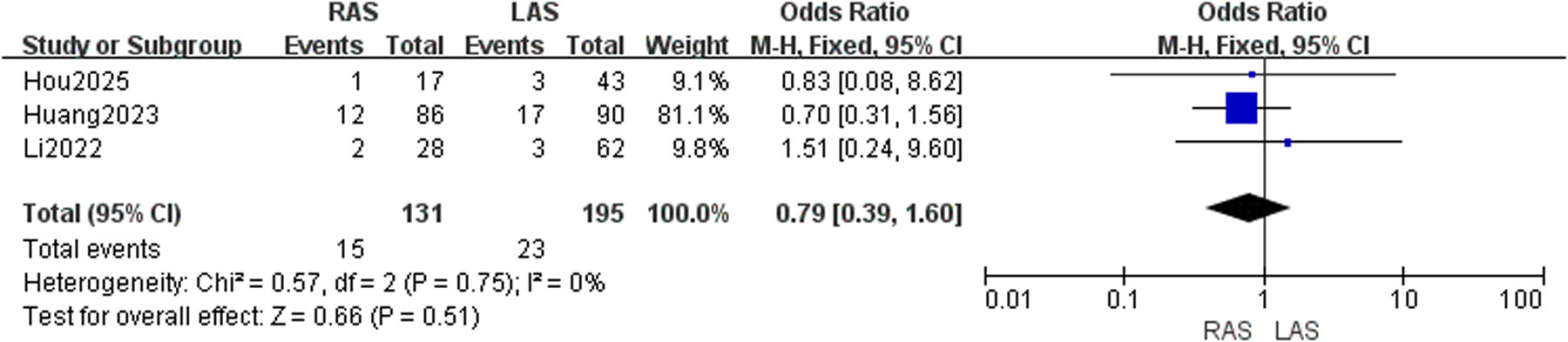
Forest plot of urinary retention rate.

Two studies (20, 24) involving 504 patients showed moderate heterogeneity (*I*^2^ = 64%, *P* = 0.10). Random-effects model revealed no significant difference (OR = 0.23, 95%CI: 0.01–3.59, *P* = 0.29) (Figure 11).

#### Gastrointestinal function recovery time

**Figure 12:**
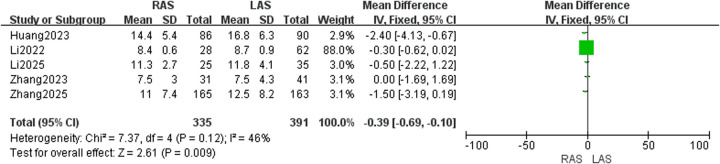
Forest plot of gastrointestinal function recovery time.

Three studies (22, 23, 25) involving 225 patients showed high heterogeneity (*I*^2^ = 96%, *P* < 0.00001). Random-effects model found no significant difference (OR = −1.27, 95%CI: −3.70–1.15, *P* = 0.30) (Figure 12).

#### Hospital stay

**Figure 13:**
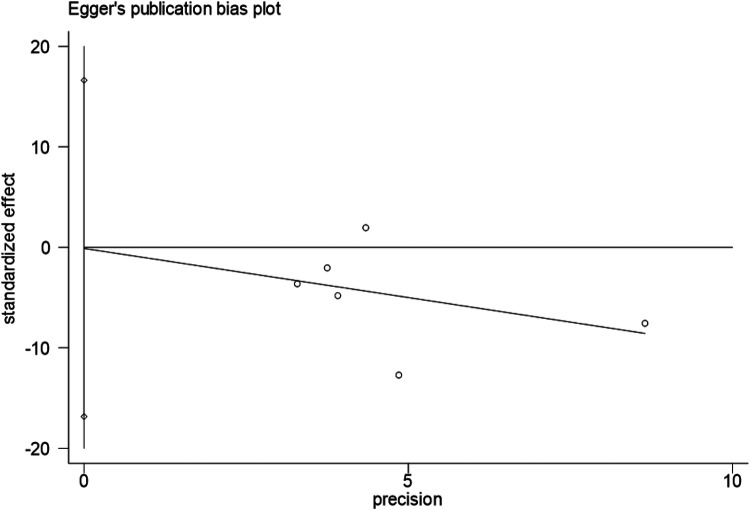
Forest plot of hospital stay.

Five studies (20, 21, 23–25) included 335 RAS and 391 LAS patients. Fixed-effects model (*I*^2^ = 46%, *P* = 0.12) showed significantly shorter hospital stay in the RAS group (OR = −0.39, 95%CI: −0.69–−0.10, *P* = 0.009) (Figure 13).

#### Publication bias

**Figure 14:**
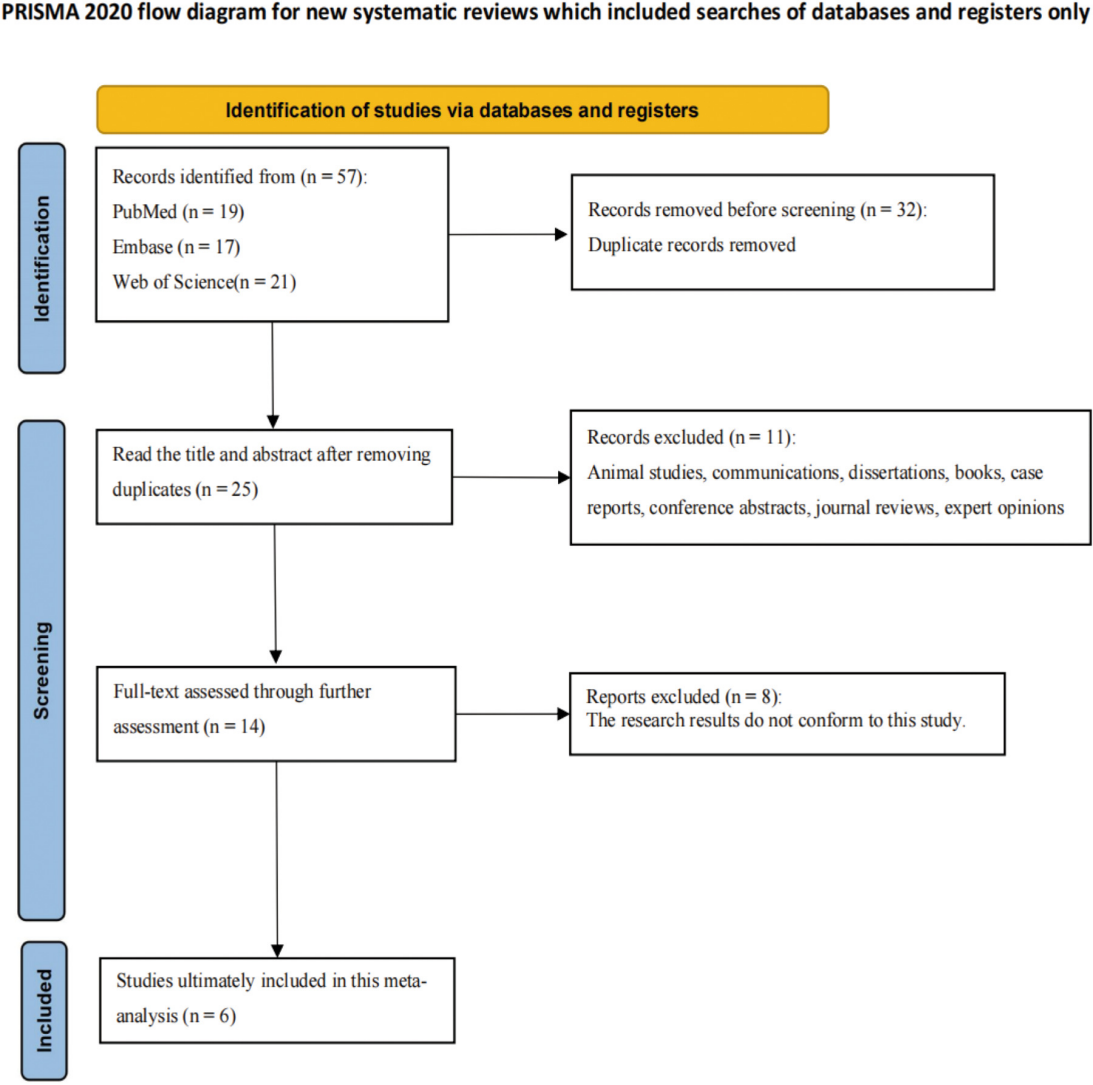
Egger's test result for publication bias.

**Figure 15:**
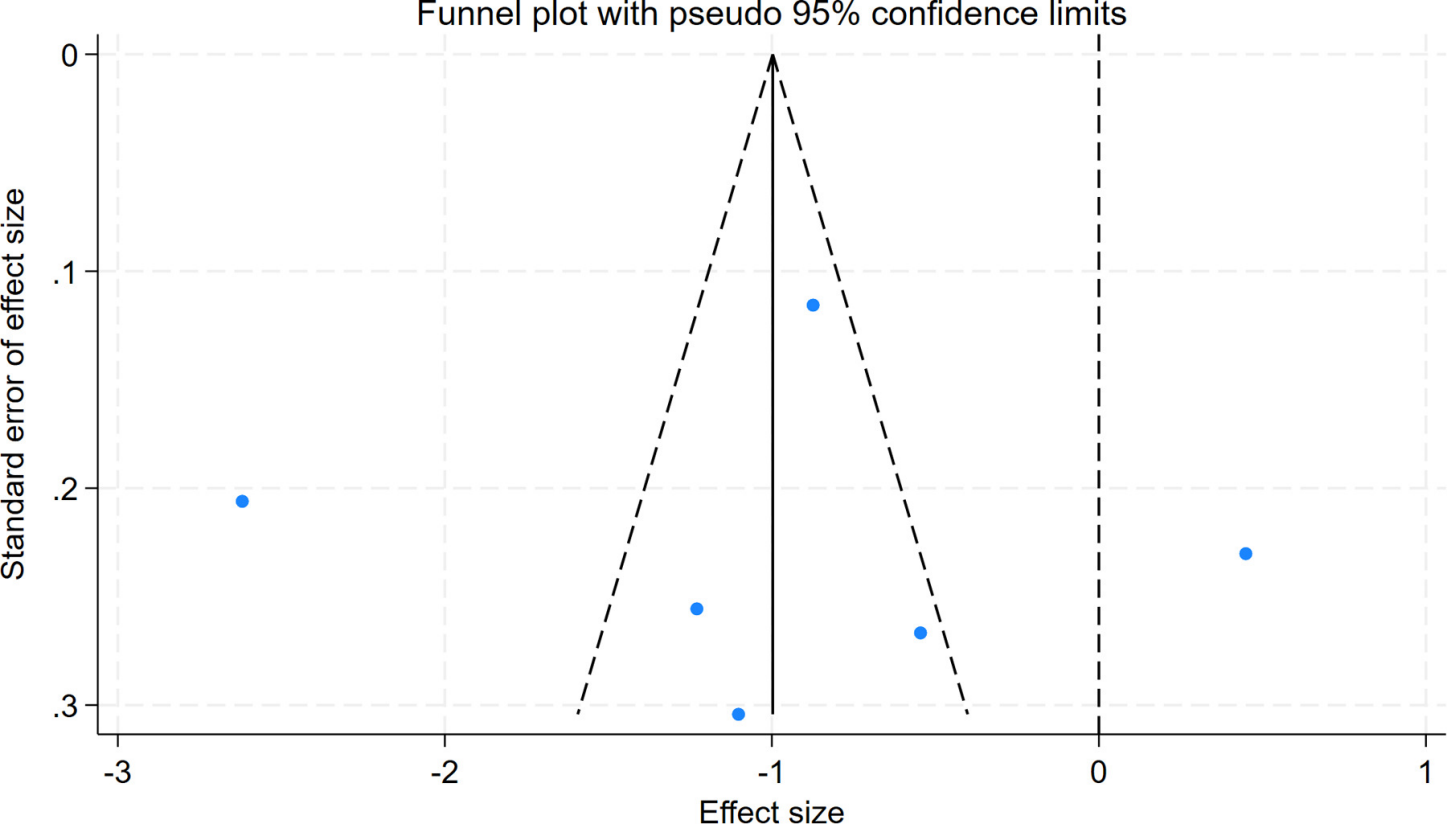
Funnel plot of publication bias.

Egger's test for intraoperative blood loss indicated no significant publication bias (*P* = 0.987), with symmetric funnel plot distribution observed (Figures 14,15).

## Discussion

This meta-analysis, the first to systematically compare outcomes of RAS and LAS for pediatric HSCR, integrated data from six cohort studies (789 patients). Results showed that RAS significantly reduced intraoperative blood loss (OR = −6.45, 95%CI: −11.77 to −1.14, *P* = 0.02) and hospital stay (OR = −0.39, 95%CI: −0.69 to −0.10, *P* = 0.009). However, longer operative duration was observed in the RAS group (OR = 19.74, 95%CI: 1.75–37.72, *P* = 0.03), with no significant intergroup differences in postoperative complications or gastrointestinal function recovery time.

The significant reduction in intraoperative blood loss with RAS is likely attributed to its three-dimensional high-definition vision and precise manipulation via multi-degree-of-freedom robotic arms. The robotic surgical system's stereoscopic imaging enhances visualization of anatomical relationships between the aganglionic bowel segment and surrounding neurovascular structures. This, combined with robotic arm flexibility, enables surgeons to dissect pathological tissues more accurately, minimizing traction or injury to blood vessels and nerves and reducing intraoperative blood loss (26, 27). The minimally invasive nature of RAS may also accelerate postoperative recovery, enabling earlier fulfillment of discharge criteria. This mechanism could explain the shorter hospital stay and reduced risk of nosocomial infections.

Several factors contribute to the longer operative duration in the RAS group. First, robotic arm installation, debugging, and positioning require additional time, especially during the early learning phase when workflows are less optimized. Second, the high precision required for robotic surgery may necessitate more time for delicate maneuvers during critical steps, such as neurovascular dissection or anastomosis. Additionally, the robotic system's learning curve and teamwork coordination between surgeons and the operative team may affect overall surgical efficiency.

The absence of significant differences in prognostic outcomes between the two groups may be ascribed to multiple factors. Laparoscopy, a mature technique in major tertiary hospitals, has undergone technical homogenization, which is likely to have narrowed the performance gap with RAS. Moreover, the limited sample size (6 studies involving 789 patients) may have reduced statistical power, thereby elevating the risk of false-negative results. Confounding factors, such as inconsistent preoperative bowel preparation and variations in perioperative antibiotic use across studies, may also have obscured true differences. Owing to the small number of included studies and limited number of participants, the conclusions of this study related to postoperative outcomes, including enterocolitis, soiling, urinary retention, and adhesive intestinal obstruction, should be interpreted cautiously. Nevertheless, they can serve as a reference for research directions.

Regarding cost-effectiveness, limited data are available on the costs of RAS and total hospitalization expenses. However, brief descriptions of surgical costs alone and total hospitalization costs have been provided by Hou et al. (22) and Huang et al. (24), respectively. Specifically, the surgical cost for RAS has been reported as $3,758.40, with a total hospitalization cost of $9,145.44. In contrast, LAS has a sugical cost of $278.40 and a total hospitalization cost of $5,945.24. It is evident that the current treatment cost of RAS is relatively high, which may affect the acceptance of RAS among families of affected children.

This study offers two key innovations. First, it is the first meta-analysis to comprehensively evaluate multiple complications of RAS and LAS for pediatric HSCR. Second, by assessing intraoperative metrics, postoperative complications, gastrointestinal recovery time, and hospital stay, it provides multidimensional evidence for clinical decision-making. However, limitations include the small number of included studies and potential residual confounding inherent to observational research. Notably, no RCTs comparing RAS and LAS for HSCR have been published to date.

Future research should focus on large-sample, long-term multicenter RCTs to eliminate biases in observational studies and validate the long-term benefits of RAS. Integration of intelligent technologies, such as artificial intelligence-assisted preoperative imaging for automated surgical path planning, may enhance robotic surgery precision. Although cost was not addressed in this study due to limited data, the economic burden of RAS on families warrants attention in future cost-effectiveness analyses.

In summary, this study provides evidence supporting the clinical application of RAS in the surgical management of pediatric HSCR. Its advantages, including reduced intraoperative blood loss and shorter hospital stays, merit attention. However, the current data have limitations, as only 6 studies with relatively small sample sizes were included. Definitive conclusions on the long-term prognosis and cost-effectiveness of RAS remain elusive. Further research is needed for validation. Meanwhile, continuous optimization of technical protocols and exploration of cost-control strategies are critical to fully utilizing robotic surgery's role in enhancing patient outcomes.

## Data Availability

The original contributions presented in the study are included in the article/Supplementary Material, further inquiries can be directed to the corresponding author.

## Supplementary material

Supplementary Table S1 Basic characteristics and quality scores of included studies.
