# Supplementary material for: Comparison of robotic-assisted and laparoscopic-assisted surgery in the treatment of children with Hirschsprung's disease: a systematic review and meta-analysis
Source: Front Pediatr. 2025 Aug 6;13:1638198. doi: 10.3389/fped.2025.1638198 (PMC12364639; doi:10.3389/fped.2025.1638198)
Supplement: Supplementary Table S1 — Basic characteristics and quality scores of included studies. [file Table1.docx]

| Author | Year | Study Type | Country | Total Sample Size (Male:Female) | Age (Months) | Follow-up Duration (Months) | Surgical Techniques | Intraoperative Metrics | Complication Categories | NOS Score |
| --- | --- | --- | --- | --- | --- | --- | --- | --- | --- | --- |
| Li | 2022 | Retrospective cohort | China | 90 (60:30) | <36 | 32 | Modified Soave | Blood loss, operative duration | ①②③④⑦⑨⑩ | 8 |
| Huang | 2023 | Retrospective cohort | China | 176 (110:66) | 8.2 | 24 | Soave | Blood loss, operative duration | ①②③④⑤⑧⑨⑩ | 8 |
| Zhang | 2023 | Retrospective cohort | China | 75 (60:15) | 9.5 | 19.3 | Swenson | Blood loss, operative duration | ①②⑩ | 8 |
| Li | 2025 | Retrospective cohort | China | 60 (42:18) | 35.2 | 3 | Modified Soave | Blood loss, operative duration | ①②⑥⑦⑩ | 7 |
| Hou | 2025 | Retrospective cohort | China | 60 (53:7) | 1.2 | 20 | Swenson | Blood loss, operative duration | ①②③⑤ | 8 |
| Zhang | 2025 | Prospective cohort | China | 328 (224:104) | ≤3 | 12 | Soave | Blood loss, operative duration | ①②⑤⑥⑦⑧⑩ | 8 |

Note. ① Enterocolitis; ② Anastomotic complications; ③ Soiling; ④ Adhesive intestinal obstruction; ⑤ Wound infection; ⑥ Incisional hernia; ⑦ Perianal infection; ⑧ Urinary retention; ⑨ No complications; ⑩ Gastrointestinal function recovery time; NOS score: Newcastle-Ottawa Scale.
